# Preliminary Study on Polishing SLA 3D-Printed ABS-Like Resins for Surface Roughness and Glossiness Reduction

**DOI:** 10.3390/mi11090843

**Published:** 2020-09-08

**Authors:** Jungyu Son, Hyunseop Lee

**Affiliations:** 1 Department of Mechanical System Engineering, Tongmyong University, Busan 48520, Korea; ccczczc@naver.com; 2 School of Mechanical Engineering, Tongmyong University, Busan 48520, Korea

**Keywords:** chemical mechanical polishing (CMP), 3D printing, stereolithography apparatus (SLA), acrylonitrile butadiene styrene (ABS)-like resin, surface roughness, glossiness

## Abstract

After the development of 3D printing, the post-processing of the 3D-printed materials has been continuously studied, and with the recent expansion of the application of 3D printing, interest in it is increasing. Among various surface-machining processes, chemical mechanical polishing (CMP) is a technology that can effectively provide a fine surface via chemical reactions and mechanical material removal. In this study, two polishing methods were evaluated for the reduction of surface roughness and glossiness of a stereolithography apparatus (SLA) 3D-printed ABS (acrylonitrile butadiene styrene)-like resin. Experiments were conducted on the application of CMP directly to the 3D-printed ABS-like resin (one-step polishing), and on the application of sanding (#2000) and CMP sequentially (two-step polishing). The one-step polishing experiments showed that it took a considerable period of time to remove waviness on the surface of the as-3D printed specimen using CMP. However, in the case of two-step polishing, surface roughness was reduced, and glossiness was increased faster than in the case of one-step polishing via sanding and CMP. Consequently, the experimental results show that the two-step polishing method reduced roughness more efficiently than the one-step polishing method.

## 1. Introduction

Chemical mechanical polishing (CMP) is a hybrid machining process that flattens the surface of a material by chemical surface reactions and a mechanical material removal method using abrasive particles located on the real contact area (RCA) between a polishing pad and the material to be polished [1,2,3,4,5,6,7]. CMP is mainly used to reduce the surface roughness of electronic materials for semiconductors, and among machining processes, it is one of the most effective process used in reducing the surface roughness of materials [2]. Recently, researchers have applied CMP to the surface processing of various materials in various fields [8]. In particular, CMP can be applied to the surface processing of polymeric materials as well as silicon, metal, and oxide films for semiconductors, which are the main targets for processing. In CMP, polyurethane pads or polyurethane impregnated pads are mainly used, and grooves are formed on the surface of the pad to facilitate a slurry flow [9,10,11]. The CMP process employs slurry with different chemical compositions, and abrasive particles depending on the target material [12,13,14,15]. Therefore, research on material removal methods for various materials is essential.

Three-dimensional (3D) printing is an additive manufacturing (AM) technology that can be used to produce three-dimensional parts, and has recently been applied to various industries such as construction, apparel, dentistry, electronics, automotive, etc. [16,17]. It is generally difficult to avoid having rough surfaces on 3D-printed parts because they are manufactured using a layer by layer printing method. There are several types of AM methods such as fused deposition modeling (FDM), stereolithography apparatus (SLA), digital light processing (DLP), and selective layer sintering (SLS) [18,19,20,21]. Optically transparent 3D printing materials, in particular, can be used for automatic lenses, bottles, and light pipes [22]. Optically transparent 3D printing materials have also recently been used to visualize fluid flow in microfluidic systems [23,24]. However, it is still necessary to improve transparency via improved surface roughness.

Yang et al. [25] said that the problem of 3D-printed products’ poor surface finish is generally caused by “stair stepping” from the principle of AM, and its application is limited due to part accuracy and performance problems. Studies to reduce surface roughness of SLA 3D-printed parts include research on the parameters of software and hardware in pre-processing [25,26,27,28] and the study on finishing and coating techniques in post-processing [29,30]. Williams and Melton [29] demonstrated the application of abrasive flow machining (AFM) in post-processing of 3D-printed parts. Ahn and Lee [30] proposed a combined surface finishing method using coating and grinding processes.

The research on the material removal of polymer materials in CMP was mainly carried out for the purpose of forming structures in micro-electro mechanical systems (MEMS) and integrated circuits (ICs). Neirynck et al. [31] studied a surfactant for a polymer CMP slurry. They explained that the polymer becomes soluble in the slurry due to the adsorption of surfactant molecules, thus increasing the material removal rate (MRR) of the polymer in the CMP when using a slurry containing a surfactant. Zhong et al. [32] investigated CMP and poly-methyl-methacrylate (PMMA) for polycarbonate (PC) and MEMS fabrication, respectively. In their study, commercial CMP pads and slurries were applied to PC and PMMA CMP, and the slurry containing ammonium hydroxide (NH_4_OH) and fumed silica exhibited the highest MRR. However, in terms of surface roughness, colloid silica slurry exhibited high efficiency. Zhong et al. [33] also studied the changes in MRR according to the applied pressure and rotating speed in PMMA and PC CMP. Towery and Fury [34] studied the CMP slurry for poly(arylene). Based on the results of basic experiments using various kinds of abrasives and oxidizers, they used a slurry containing Fe(NO_3_)_3_ as an oxidant, and fumed silica (175 nm) as an abrasive in CMP for poly(arylene) ether, indicating that MRR increases as the concentration of abrasive particles increases. Lee et al. [35] proposed a way to chemical-mechanically polish the thick Cu film and negative photoresist (PR) in MEMS at the same time, after which the MRRs of copper and negative PR were measured. In their study, a commercial acidic copper CMP slurry containing an oxidizer, complexing agent, surfactant, corrosion inhibitor, and colloidal silica particles was used in the CMP experiment. Although studies are being conducted on CMP for polymer materials, few CMP studies are being conducted on polymer materials for 3D printing.

In this study, a preliminary study was conducted on the surface roughness and glossiness reduction of transparent SLA 3D-printed acrylonitrile butadiene styrene (ABS)-like resin material. A one-step polishing process that employs CMP immediately after 3D printing was compared with a two-step polishing process that uses sanding and CMP sequentially.

## 2. Experimental Setup

### 2.1. Specimens 3D Printing Methodology

**Figure 1:**
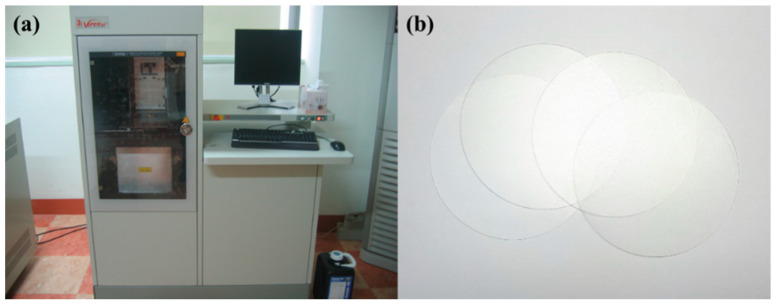
Three-dimensional (3D) printer and 3D printed discs: (**a**) stereolithography apparatus (SLA) 3D printer (SLA-si2) used in this study (**b**) SLA 3D printed acrylonitrile butadiene styrene (ABS)-like resin discs.

A Viper SLA-si2 (3D Systems, Rock Hill, SC, USA) 3D printer (Figure 1a) was used to fabricate specimens in the polishing experiments. In basic polishing experiments, a disc, with a diameter of 100 mm and thickness of 1 mm, was 3D-printed (Figure 1b). The printing material used was WaterClear^®^ Ultra 10122 from DSM Somos^®^ [22], a resin with ABS-like properties. The discs were 3D-printed vertically (to minimize deformation) and the layer thickness of the printing was 0.1 mm with solid structure.

The average weight of the 3D printed disc was 8.3323 g, and the calculated density of 3D-printed resin was 1.06 g/cm^3^ (the density of the material datasheet provided by SDM Somos^®^ is approximately 1.13 g/cm^3^). The measured average hardness (Shore D) of the 3D-printed specimen (disc) was 81.1 after 3D-printing.

### 2.2. Polishihng Methodology

A G&P POLI-300 polisher (G&P Technology, Busan, Korea) was used in the polishing experiments. These experiments were conducted in two case methods: one-step and two-step polishing methods. Table 1 shows the two cases of the polishing methods. A hard polyurethane pad (KONI pad from KPX Chemical, Seoul, Korea) with good planarization properties was selected for the planarization of the surface. And, a colloidal silica slurry were used for the CMP experiments. The average SiO_2_ particle size of the slurry used was 72.0 nm while the initial concentration of SiO_2_ particles was 40 wt%. In the experiment, the slurry was diluted 1:1 with deionized water (DIW), and the concentration of particles after dilution was 20 wt%. The applied pressure, rotating speed of the head and platen, and slurry flow rate were 41.2 kPa, 150 rpm, and 150 mL/min, respectively. A piezoelectric quartz sensor (Kistler Type 9135B) was mounted on the back of the polishing head to measure the dynamic frictional force during CMP. Pad conditioning was performed every 10 min in the CMP. Table 2 shows the experimental conditions of CMP.

In the two-step polishing case, CMP was performed after the disc was polished for 2 min using sandpaper (#2000) with some DIW. Polishing was applied using sandpaper to quickly remove the waviness on the surface of the material owing to 3D printing. The applied pressure and rotating speed of the disc in sanding were 9.81 kPa and 80 rpm, respectively. After sanding and CMP were applied, the disc was cleaned with PVA brush scribing and dried in dry air.

### 2.3. Measurement of Surface Roughness and Glossiness

**Figure 2:**
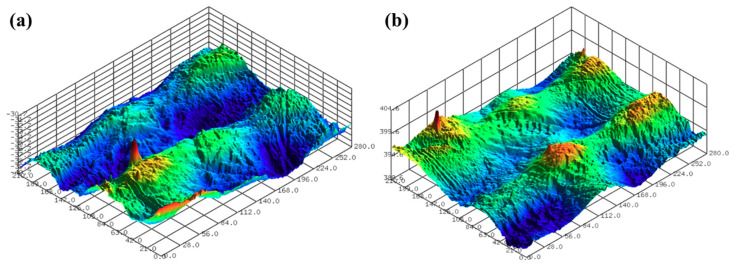
Surface profiles after SLA 3D printing; (**a**) Sa of 1.002 μm and Sz of 8.947 μm, and (**b**) Sa of 1.065 μm and Sz of 9.401 μm.

The surface roughness of the 3D-printed disc was measured using a confocal laser microscope (NS-3500, NANOSCOPE SYSTEMS Inc., Daejeon, Korea). Figure 2 shows the 3D surface images of the 3D-printed discs. The average arithmetical mean deviation (Sa) and average maximum height (Sz) values of the 3D printed specimens used in the experiments were 1.432 μm and 9.720 μm, respectively. Five positions were measured in the radial direction from the center of the disc to the up, down, left, and right, and the interval between the measurements was 20 mm.

**Figure 3:**
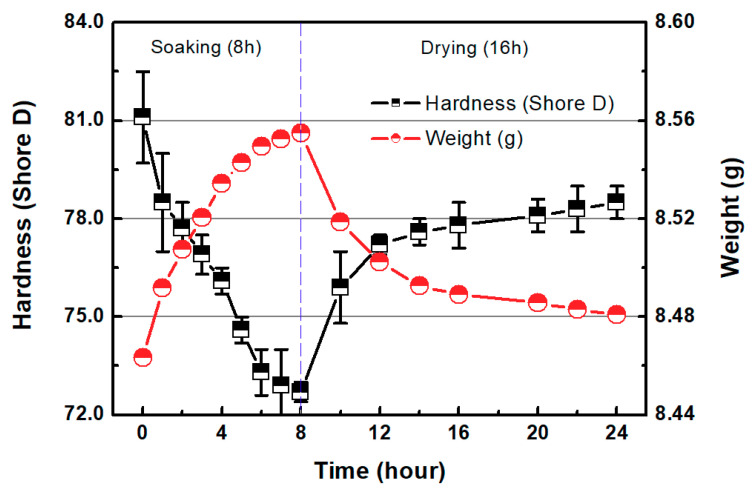
Hardness and weight of SLA 3D-printed ABS-like resin as functions of soaking and drying time.

In general, the MRR of a transparent material is calculated using the weight loss and material density in CMP. However, the SLA 3D-printed resin absorbs water when exposed to humid environments, as shown in Figure 3. Figure 3 shows the weight and hardness of the 3D-printed disc after soaking it in water (1 L) for 8 h and allowing it to dry in air (at room temperature) for 16 h using an electronic precision balance (0.1 mg resolution) and a digital Shore D hardness tester (LX-D-Y/D-Type, TRIPOD, China) for hard rubber and plastic. While soaking the disc, its weight increased over time, whereas its Shore D hardness gradually decreased. This phenomenon seems to occur because resin absorbs water and reacts chemically as water penetrates into fractures or cavities in the material [36,37]. These results demonstrate the difficulty of measuring MRR in polished SLA 3D-printed ABS-like resins. Therefore, in this study, the surface roughness and glossiness of the 3D-printed material are presented as a result of polishing. Glossiness was measured with a gloss meter (NHG268, 3nh^®^, China) in gloss units (GU). The measurement of glossiness followed an ISO 2813 standard. In this study, the glossiness of the as-3D printed ABS-like resins was 14.23 ± 1.67 GU. The measurements of glossiness were taken at five points on the specimen. A field-emission scanning electron microscope (FE-SEM, Quanta 200, Thermo-Fisher-Scientific, Waltham, MA, USA) was used for further surface observation.

## 3. Results and Discussion

### 3.1. One-Step Polishing

**Figure 4:**
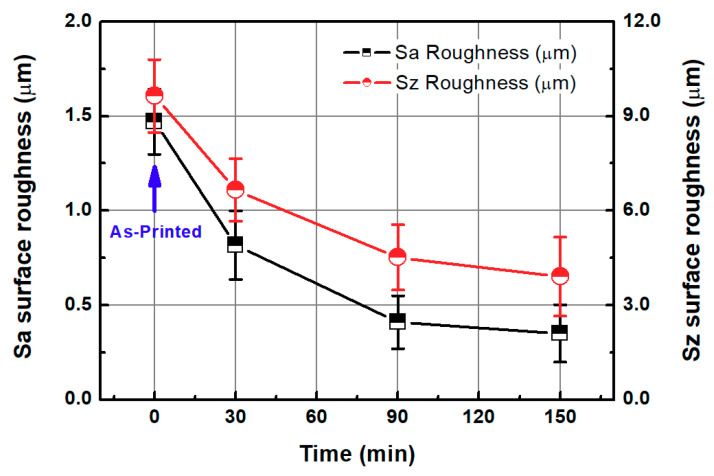
Sa and Sz values as functions of CMP processing time in one-step polishing method.

**Figure 5:**
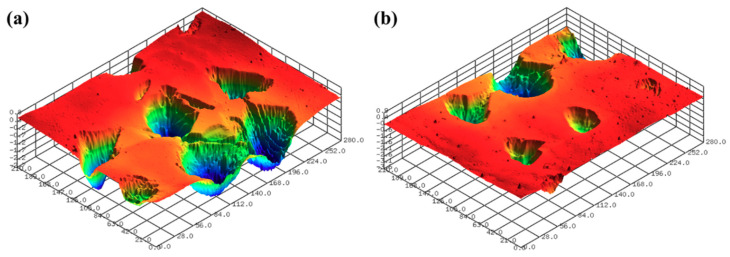
Representative surface profiles of SLA 3D-printed ABS-like resin after CMP for 150 min; (**a**) Sa of 0.490 μm and Sz of 3.805 μm (**b**) Sa of 0.393 μm and Sz of 3.755 μm.

**Figure 6:**
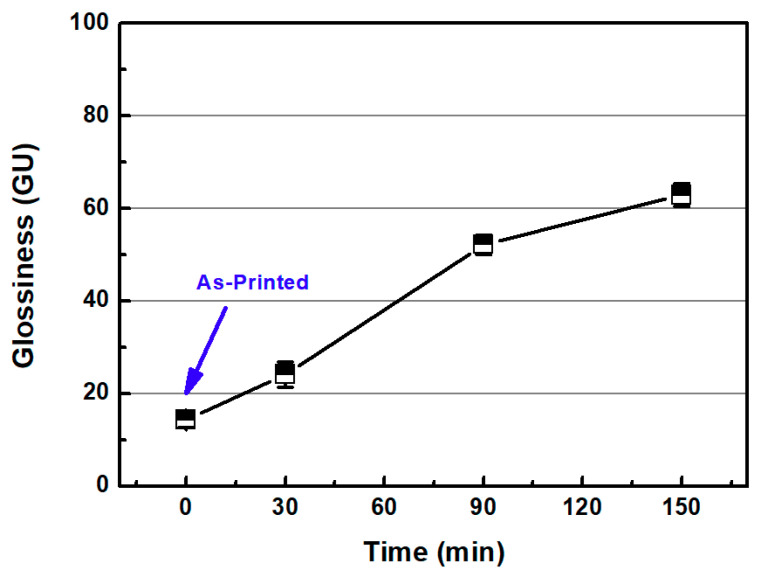
Glossiness as a function of CMP processing time with one-step polishing method.

In this section, the roughness reduction characteristic of the 3D-printed disc was examined by a CMP experiment. The 3D-printed layers of resin can be found on the surface of the 3D-printed disc, as shown in Figure 2. In this experiment, the average Sa and Sz values of the 3D-printed disc were 1.683 μm and 11.117 μm, respectively. Deep valleys were observed on the surface of the specimen, and to improve the roughness and glossiness conditions of the surface, waviness and fine roughness on the surface must be removed at the same time. In this study, a hard pad with good planarization properties was selected for the planarization of the surface, and a colloidal silica slurry was used for the CMP experiment. The applied pressure and rotating speed were 41.2 kPa and 150 rpm, respectively, as shown in Table 2. Figure 4 shows Sa and Sz values over the processing time of CMP in the one-step polishing method. The Sa value decreases from 1.470 μm to 0.350 μm after 150 min of CMP. The Sz value also decreased from 9.637 μm to 3.907 μm after 150 min. After starting the CMP, the surface roughness continued to decrease until 90 min, but after 90 min, the surface roughness was not significantly reduced by the CMP. Figure 5a,b show the representative surface profiles of the SLA 3D-printed ABS-like resin after CMP was applied for 150 min. Figure 5 shows that only the top part of the waviness on the surface of the specimen is removed and flattened. The glossiness of the as-3D printed ABS-like resin in Figure 6 was 14.28 GU, but it increased to 62.94 GU after 150 min of polishing. The results of the experiment indicate that it takes a considerable period of time to secure a flat surface via polishing the SLA 3D-printed ABS-like resins using the one-step polishing method.

As mentioned earlier, the ABS-like resin used in the experiment has a water-absorbing property. Resin absorbs water and reacts chemically with water to cause the hydrolysis of resin, resulting in the scission of the chain as follows [38]:

(1) $$\sim A-B\sim +H_{2}O\;\to \sim A-OH+\sim B-H$$

where *A* and *B* are chemical groups in the ABS-like resin.

The absorption characteristics of 3D-printed ABS-like resin appear to reduce hardness, making it easier to reduce surfaces roughness.

**Figure 7:**
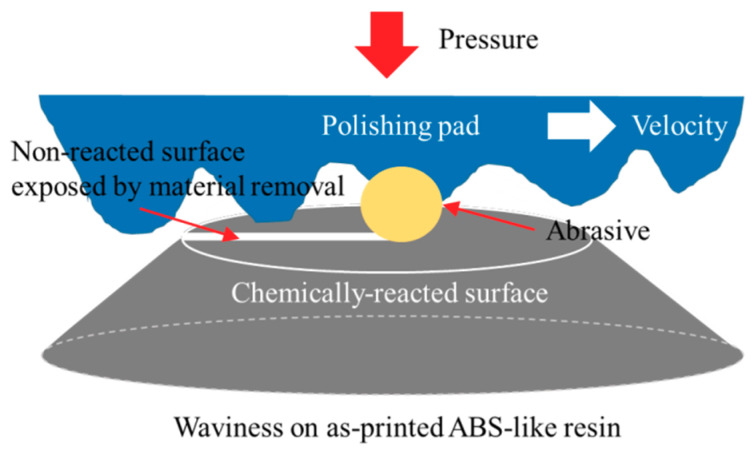
Schematic diagram of ABS-like resin CMP in one-step polishing.

As shown in Figure 3 and Equation (1), the hydrolysis of the water-absorbing ABS-like resin reduced hardness, making it easier to remove materials via CMP. Figure 7 shows a schematic diagram of the material removal of the ABS-like resin CMP in one-step polishing. The abrasives in the CMP slurry mechanically can feasibly remove materials on the surface of the hydrated ABS-like resin. The deep valley in the 3D-printed ABS-like resin appears difficult to remove with CMP alone owing to its low MRR. Therefore, in order to planarize the surface of the SLA 3D-printed ABS-like resin, CMP should be preceded by a process that can remove surface waviness faster.

### 3.2. Two-Step Polishing

#### 3.2.1. Sanding

As shown in the experiment conducted in Section 3.1, it can be inferred that a considerable period of time is required to immediately remove the waviness of the 3D-printed disk surface using CMP. In this section, the authors suggest that sanding and CMP should be carried out sequentially to planarize the surface of the 3D-printed ABS-like resin. The average Sa and Sz values of the ABS-like resin specimens used in the experiment were 1.394 μm and 9.803 μm, respectively. The sanding process was carried out for 2 min to eliminate surface waviness quickly using sandpaper (#2000) together with some DIW. The pressure and rotating speed were 9.81 kPa and 80 rpm, respectively, as shown in Table 2.

**Figure 8:**
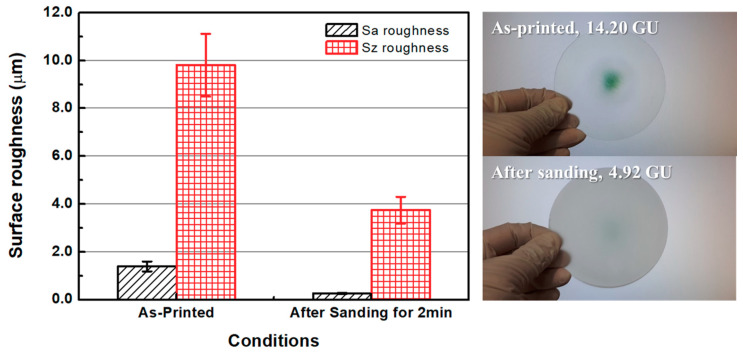
Surface roughness and glossiness of 3D-printed ABS-like resin before and after sanding.

**Figure 9:**
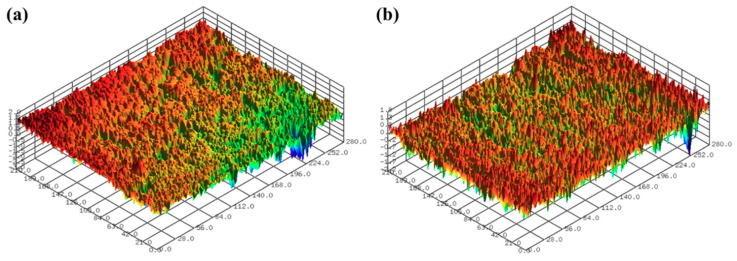
Representative surface profiles of SLA 3D-printed ABS-like resin after sanding for 2 min; (**a**) Sa of 0.256 μm and Sz of 3.939 μm (**b**) Sa of 0.241 μm and Sz of 3.523 μm.

**Figure 10:**
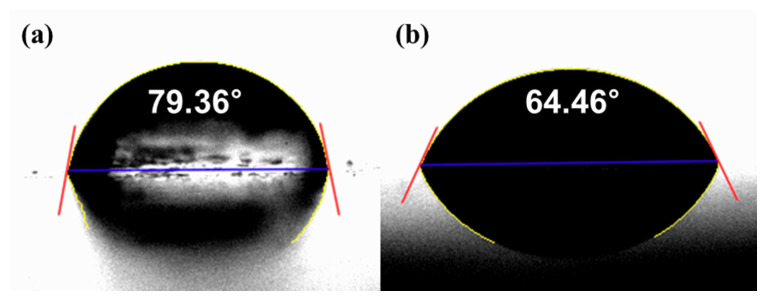
Contact angle between DIW and ABS-like resin; (**a**) after 3D-printing and (**b**) after sanding for 2 min.

Figure 8 shows the surface roughness values of the as-3D printed ABS-like resin before and after sanding. The initial average Sa and Sz values of the specimen were 1.394 and 9.803 μm, respectively, as mentioned earlier. After 2 min of sanding, these Sa and Sz values were reduced to 0.266 μm and 3.744 μm, respectively. Glossiness was 14.20 GU after 3D printing, but was lowered to 4.92 GU after sanding. Figure 9 shows the results of measuring the specimen with a confocal laser microscope after sanding, including its representative surface profiles. It is evident that the waviness of the surface formed by 3D printing was removed by sanding. Despite the decrease in surface roughness after sanding, micro-roughness was formed on the surface of the specimen, which appeared to reduce glossiness and transparency. The pictures on the right in Figure 8 show that the transparency of the specimen deteriorated after sanding. Moreover, the wettability of water for ABS-like resin was increased by sanding, as shown in Figure 10. The as-3D printed ABS-like resin had a contact angle of 79.36° (Figure 10a), but its contact angle decreased to 64.46° (Figure 10b) after sanding. In previous research, it was reported that the change in surface roughness has an effect on wettability [39,40]. High wettability may have the effect of increasing the probability of slurry participating in material removal during CMP.

#### 3.2.2. Chemical-Mechanical Polishing (CMP) after Sanding

**Figure 11:**
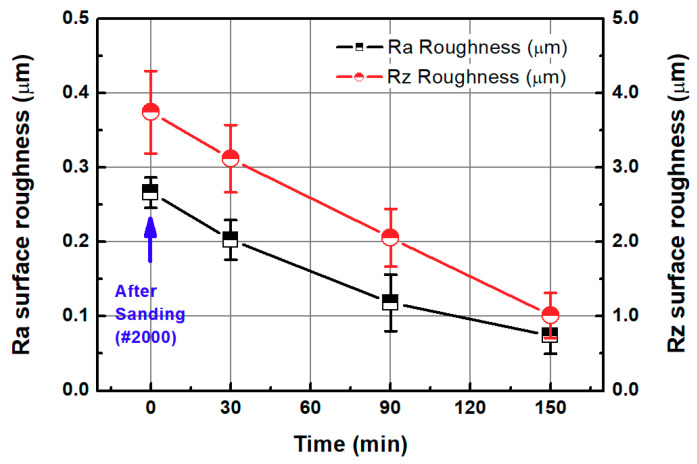
Sa and Sz values as functions of CMP processing time in two-step polishing method.

After sanding the 3D-printed ABS-like resin, fine roughness was removed by the CMP. The process conditions of the CMP are shown in Table 2 and Section 3.1. In Figure 11, the Sa surface roughness of the specimen after sanding was 0.266 μm but decreased to 0.073 μm via a 150-min CMP. After sanding, the Sz surface roughness of the specimen decreased from 3.744 μm to 1.012 μm after 150 min of CMP.

**Figure 12:**
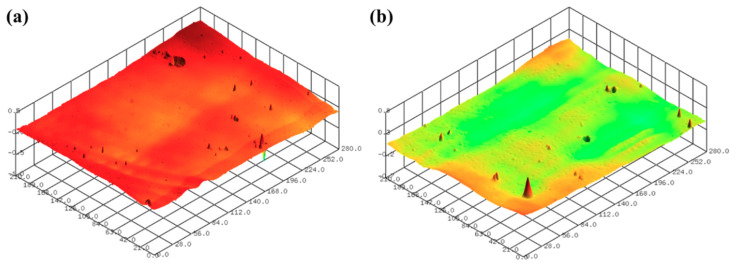
Representative surface profiles of SLA 3D-printed ABS-like resin after sanding and CMP for 2 min and 150 min, respectively; (**a**) Sa of 0.041 μm and Sz of 1.281 μm (**b**) Sa of 0.037 μm and Sz of 1.135 μm.

**Figure 13:**
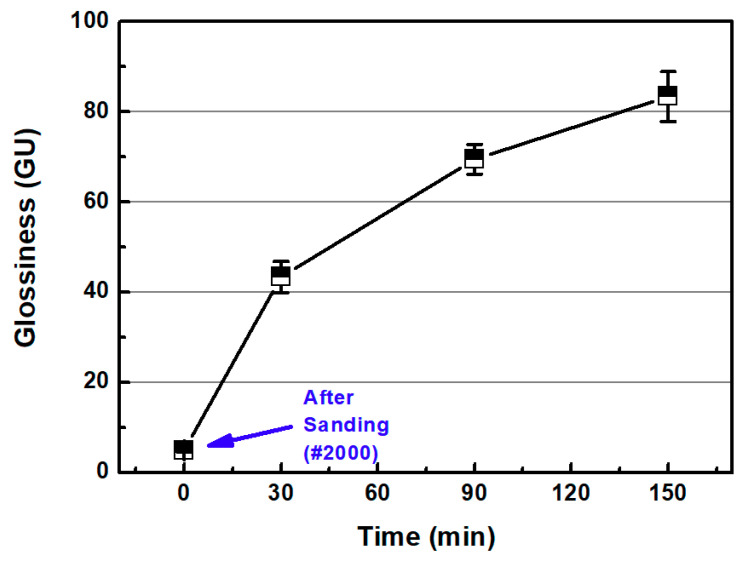
Glossiness as a function of CMP processing time with two-step polishing method.

Figure 12a,b show the representative surface profiles of the SLA 3D-printed ABS-like resin after sanding and CMP for 2 min and 150 min, respectively. After sanding with sandpaper (#2000), the glossiness of the ABS-like resin was 4.92 GU. The glossiness of the resin increased from 4.92 GU to 83.38 GU in accordance with CMP processing time (Figure 13). In the one-step polishing method, glossiness was 62.94 GU after 150 min, but in the two-step polishing method, the glossiness after CMP increased to 83.38 GU. The two-step polishing method, which consists of the sequential process of sanding and CMP, shows higher processing efficiency than the one-step polishing method because it involves quickly removing the waviness formed on the surface after 3D printing via sanding, and removing fine surface asperities by CMP. In CMP, this may be because it is easier to perform material removal on the damaged layer left on the surface of the material by sanding.

### 3.3. Comparison of One-Step and Two-Step Polishing Methods

The one-step and two-step polishing methods are compared in terms of surface roughness reduction. To evaluate the performance of each method at reducing surface roughness, the roughness reduction efficiency was defined as follows:

(2) $$Roughness\;reduction\;efficiency=\;\frac{\left(Initial\;roughness-Final\;roughness\right)}{Initial\;roughness\;\times \;Processing\;time}\times 100$$

Generally, in the case of polishing, the greater the surface roughness of the specimen surface, the higher the material removal rate. Therefore, the roughness reduction efficiency in this study is represented as the ratio of surface reduction rate to the initial surface roughness.

**Figure 14:**
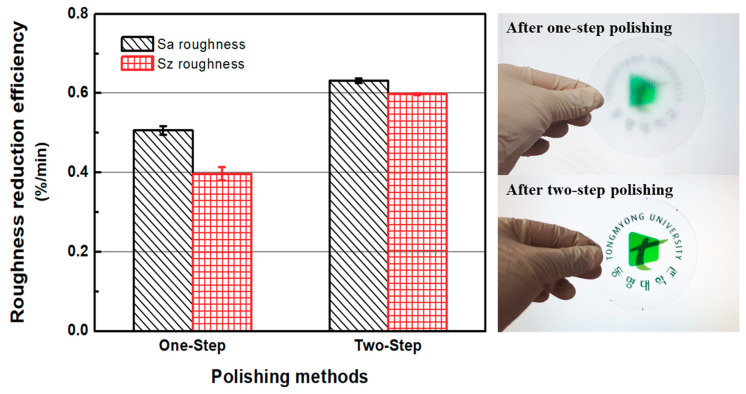
Roughness reduction efficiencies of one-step and two-step polishing methods and pictures of ABS-like resin disks after one-step polishing and two-step polishing (polished on both sides).

Figure 14 shows the roughness reduction efficiencies of the one-step and two-step polishing methods. Overall, it is evident that with sanding and CMP, the efficiency of the two-step polishing method is higher than that of the one-step polishing method. In the one-step polishing method, the roughness reduction efficiencies of Sa and Sz were 0.506%/min and 0.397%/min, respectively. The Sa and Sz roughness reduction efficiencies in the two-step polishing method were 0.632%/min and 0.598%/min, respectively. When compared to the one-step polishing method, the Sa and Sz roughness reduction efficiencies of the two-step polishing method were, respectively, 24.90% and 50.63% higher. The right-hand pictures in Figure 14 show ABS-like resin disks after one-step polishing and two-step polishing.

**Figure 15:**
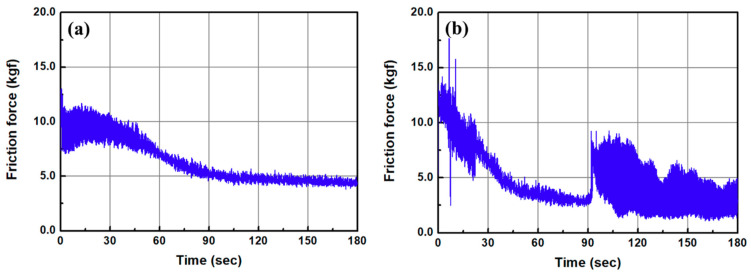
Frictional force as a function of CMP time; (**a**) after 3D printing (one-step polishing) and (**b**) after sanding (#2000) (two-step polishing).

Figure 15 shows the changes in the frictional force as a function of CMP time in the one-step (Figure 15a) and two-step polishing methods (Figure 15b). As mentioned in Section 2.2, a piezoelectric quartz sensor was mounted on the back of the polishing head to measure the dynamic frictional force. The signal from the force sensor was obtained by using a charge amplifier and data acquisition board. In the one-step polishing method, the frictional force during CMP has a high value with large variation in the early stages of processing, but over time, it decreases and stabilizes. The high frictional force in the early stage of CMP may come from the removal of fine asperities on the ABS-like resin surface, and it is stabilized as the upper part of the surface waviness is flattened. In the two-step polishing method, the frictional force decreased rapidly in the early stage of CMP, and a drastic change in the frictional force was observed after processing for approximately 92 s. This phenomenon seems to occur owing to the removal of the damaged layer of the surface after sanding, and the undamaged ABS-like resin by the CMP.

**Figure 16:**
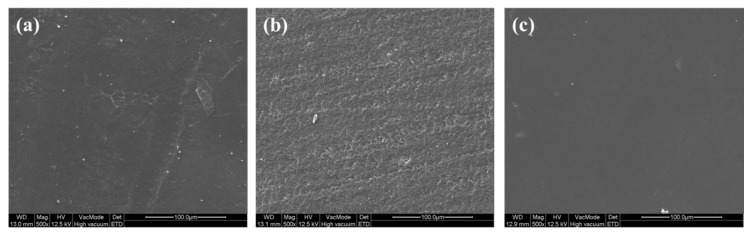
Field-emission scanning electron microscope (FE-SEM) images (×500); (**a**) as-3D printed, (**b**) after sanding, and (**c**) after sanding and CMP.

Figure 16 shows the scanning electron microscope (SEM) images (×500) of as 3D-printed ABS-like resins (a), after sanding (b), and after sanding and CMP (c). FE-SEM was used for surface observation. As shown in Figure 2 and Figure 16a, the as-3D printed sample has the waviness on its surface. The sample surface after sanding has a rough surface due to the abrasive/adhesive wear as shown in Figure 16b. If CMP is applied after sanding, abrasion marks on the surface disappear (Figure 16c).

**Figure 17:**
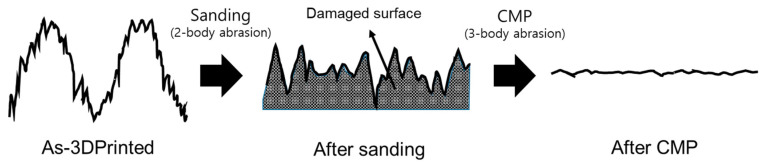
Schematic diagram of the two-step polishing mechanism.

Using 2-body abrasion, sanding process removes material by fixed abrasive and occurs abrasive/adhesive wear, leaving damages by mechanical material removal on the surface of the specimen. The generation of the damage layer on the surface of the 3D-printed ABS-like resin by the sanding process can be identified in Figure 15b as a change in friction force and in the FE-SEM image (Figure 16a). In CMP, where materials are removed through 3-body abrasion, the damaged layer produced by sanding machining is removed due to the sliding and rolling motion of abrasive particles so that a high-quality surface can be obtained. Figure 17 shows the schematic diagram of the two-step polishing mechanism.

## 4. Conclusions

In this paper, a study on the polishing methods of 3D-printed ABS-like resin materials was carried out to reduce surface roughness and increase glossiness. In the experiment, a one-step polishing method that directly applies CMP to 3D-printed materials was compared with a two-step polishing method that progresses CMP sequentially after sanding. The 3D-printed ABS-like resin had a water-absorbing property, and the hardness of the material surface was reduced with an increase in water absorption. CMP was applied to 3D-printed ABS-like resin in the one-step polishing method, and it was confirmed that the waviness or deep valley of the specimen formed by 3D printing was not completely removed after 150 min of CMP. In the two-step polishing method, the surface roughness was reduced via CMP after removing the waviness on the specimen by sanding, and compared to the one-step polishing method, the surface roughness and glossiness of the 3D-printed ABS-like resin could be improved more efficiently. The application of the sanding process prior to the CMP process seems to help remove the waviness of the 3D-printed ABS-like resin surface and leave the damaged layer on the surface, but quickly remove it through the CMP process and secure a high-quality surface roughness.

In the future, studies on the application of various abrasive particles and polishing pads in the 3D-printed ABS-like resin polishing process, studies on friction phenomena during polishing, and studies on polishing characteristics under 3D-printing conditions will be needed. In addition, the development of high-efficiency polishing processes to simplify the post-processing of 3D-printed parts and obtain high-quality surface roughing is expected to be required.

## Figures and Tables

**Table 1:** Two cases of polishing methods.

Polishing Case.	Method.
One-step polishing	Chemical mechanical polishing (CMP)
Two-step polishing	Sanding (#2000) + CMP

**Table 2:** Two cases of polishing methods.

Process.	Parameter	Value or Consumable
Sanding	Sandpaper	#2000
	Applied pressure	9.81 kPa
	Rotating speed	80 rpm
CMP	Applied pressure	41.2 kPa
	Rotating speed	Head 150 rpm/Platen 150 rpm
	Slurry flow rate	150 mL/min
	Slurry	Colloidal silica slurry (diluted with deionized water)
	Polishing pad	KONI pad (KPX Chemical, Seoul, Korea)
